# Contribution to the Study of the Horse-chestnut

**Published:** 1897-07

**Authors:** J. L. Clark

**Affiliations:** Stratford, Canada


### CONTRIBUTION TO THE STUDY OF THE HORSE-CHESTNUT.

By J. L. Clark, V.S.,
STRATFORD, CANADA.

*Æsculus Hippocastanum* (common horse-chestnut). Synonyms: *Hippocastanum Castanea folia multifido.* Though this castanea was well known to the ancients, yet Mathiolus seems to be the first author who described the horse-chestnut, which was brought into Europe about the middle of the sixteenth century, and was so scarce in the time of Clusius that there was then but one tree known at Vienna, which, being too young to bear fruit, nuts were obtained from Constantinople in 1588, after which this tree was very generally propagated. It was cultivated in England by Mr. Tradescard, in 1633, and is now very common in this country.

The wood is white, soft, soon decays, and is therefore of little value. The fruit in appearance resembles that of the Spanish chestnut, and is eaten by sheep, goats, deer, oxen, and horses. It contains much farinaceous matter, which, by undergoing a proper process, so as to divest it of its bitterness and acrimony, probably might afford a kind of bread. Starch has been made of it, and found to be very good. It appears also to possess a saponaceous quality, as it was used particularly in France and Switzerland for the purpose of cleaning woollens and in washing and bleaching linens.

With a view to its errhine power the Edinburgh College had introduced it into the materia medica. A small portion of the powder snuffed up the nostrils readily excites sneezing; even the infusion or decoction of this fruit produces this effect. It has, therefore, been recommended for the purpose of producing a discharge from the nostrils, which, in some complaints of the head and eyes, was found to be of considerable benefit.

On the Continent the bark of the horse-chestnut tree is held in great estimation as a febrifuge, and upon the credit of several reputable authors appears to be a medicine of great efficacy. Zannichelli, of Venice, was the first to publish its successful use in various cases of intermittents, since which its good effects have been confirmed by Leidenfrost, Peipers, Junghanss, Carte and Willemet, Sabarot de la Varniere, Surra, Buchholz,. and others, from whom it appears that this bark may be substituted for the Peruvian bark in every case in which the latter is indicated, and with equal if not superior advantage.

A horse-chestnut tree above eighty years old and fifty feet high has been found in a healthy and growing state. The ripe capsule seldom contains more than one nut, but on being examined in its embryo state two are constantly found. Horses are said to eat this food greedily, and by it to have been cured of coughs and pulmonary disorders, and hence the name horse-chestnut. For the purpose of fattening cattle, and particularly sheep, it has been thought necessary to macerate the nuts in caustic alkali, in order to take off the bitterness, afterward wash them in water, and then boil them to a paste. Lime-water was found to answer the purpose exceedingly well. But if the nuts are cut and mixed with oats or bran this purpose may be effected with less trouble.

The bark intended for medicinal use is to be taken from those branches which are neither very old nor very young, and to be exhibited under similar forms and doses as directed with respect to the *cortex Peruvianus*. It rarely disagrees with the stomach, but its astringent effects generally require the occasional administration of a laxative.

